# Friend or foe? A parasitic wasp shifts the cost/benefit ratio in a nursery pollination system impacting plant fitness

**DOI:** 10.1002/ece3.6190

**Published:** 2020-03-24

**Authors:** Carmen Villacañas de Castro, Thomas S. Hoffmeister

**Affiliations:** ^1^ Population and Evolutionary Ecology Group Institute of Ecology FB 02 University of Bremen Bremen Germany

**Keywords:** cost/benefit ratio, host–parasitoid interaction, plant fitness, *Silene–Hadena*–*Bracon* system

## Abstract

Nursery pollination systems are species interactions where pollinators also act as fruit/seed herbivores of the plant partner. While the plants depend on associated insects for pollination, the insects depend on the plants’ reproductive structures for larval development. The outcome of these interactions is thus placed on a gradient between mutualism and antagonism. Less specialized interactions may fluctuate along this gradient with the ecological context, where natural enemies can play an important role. We studied whether a natural enemy may impact the level of seed consumption of a nursery pollinator and how this in turn may influence individual plant fitness. We used the plant *Silene latifolia*, its herbivore *Hadena bicruris*, and its ectoparasitoid *Bracon variator* as a model plant–herbivore–natural enemy system. We investigated seed output, germination, survival, and flower production as proxies for individual plant fitness. We show that *B. variator* decreases the level of seed consumption by *H. bicruris* larvae which in turn increased seed output in *S. latifolia* plants, suggesting that parasitism by *B. variator* may act as a regulator in the system. However, our results also show that plant survival and flower production decrease with higher seed densities, and therefore, an increase in seed output may be less beneficial for plant fitness than estimated from seed output alone. Our study should add another layer to the complex discussion of whether parasitoids contribute to plant fitness, as we show that taking simple proxies such as seed output is insufficient to determine the net effect of multitrophic interactions.

## Graphical abstract

We studied whether a natural enemy may impact the level of seed consumption of a seed predator and how this in turn may influence individual plant fitness. We used the plant *Silene latifolia*, its herbivore *Hadena bicruris*, and its ectoparasitoid *Bracon variator* as a model plant‐herbivore‐natural enemy system. Although *B. variator* increases seed output in *S. latifolia* through the attack of the seed predator, next generation adult plant survival and flower production decrease with higher seed densities.

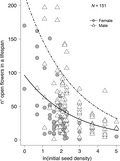

## 1 INTRODUCTION

Mutualistic interactions are fascinating examples of coevolution, in which individuals of different taxa depend on and provide benefits to each other (Boucher, James, & Keeler, 1982). Even though scientists have been studying mutualism for many decades (Micheneau, Johnson, & Fay, 2009), recent research makes it ever more evident that our understanding of the general interactions driving these processes is far from complete. For example, organisms that once were thought to cause microbial diseases are now known to act as mutualists in our gut systems, having crucial roles in our well‐being (Bäckhed, Ley, Sonnenburg, Peterson, & Gordon, 2005; Dethlefsen, McFall‐Ngai, & Relman, 2007). In another case studied by Kawakita, Mochizuki, and Kato (2015), the authors suggest that the presence of a third‐party partner (a braconid wasp) could explain the reversal of mutualism to parasitism in leaf–flower moths. A whole new world has opened up focusing on mutualism (e.g., see Dubilier, Bergin, & Lott, 2008), and there is a need to understand how these systems evolve, transform, and are maintained.

A particular type of mutualism, nursery pollination systems (sensu Dufaÿ & Anstett, 2003), has been receiving quite some attention in the past years (Kephart, Reynolds, Rutter, Fenster, & Dudash, 2006; Labouche & Bernasconi, 2013; Prieto‐Benítez, Yela, & Giménez‐Benavides, 2017; Reynolds, Fenster, Kula, & Dudash, 2012; Westerbergh, 2004). The partners in these interactions are a host plant and an insect (normally a moth, fly, or wasp) that acts as a pollinator but at the same time lays eggs in or on the plant. The offspring of the insect will then develop and feed from the reproductive structures of the plant, possibly implying high fitness costs for the plant (Dufaÿ & Anstett, 2003; Kephart et al., 2006). However, there must be a balance between the costs and benefits for both partners to achieve a positive net outcome, otherwise the mutualism would eventually turn into a parasitic interaction (Bronstein, 1994; Kawakita et al., 2015; Pellmyr, Thompson, Brown, & Harrison, 1996).

Dufaÿ and Anstett (2003) reviewed nursery pollination systems and described a total of 13 documented cases, although since then other systems have been discovered (see Kawakita & Kato, 2004; Nunes, Maruyama, Azevedo‐Silva, & Sazima, 2018; Song et al., 2014). Within these systems, there are some which are obligate mutualisms, such as the interaction between *Ficus* trees and fig wasps, *Yucca* and yucca moths, or senita cacti and senita moths (Anstett, Bronstein, & Hossaert‐McKey, 1996; Dufaÿ & Anstett, 2003; Holland & Fleming, 1999; Pellmyr et al., 1996). The insects here actively pollinate their host plant with morphological structures that increase pollen transfer and copollinators are absent from the system. This high specialization makes the system more stable and prone to a positive cost/benefit ratio. Other interactions such as the partnership between Greya moth and its host plant, *Lithophragma parviflorum*, are not specialized to that degree, lacking active pollination and having copollinators present (Thompson & Pellmyr, 1992). In the same vein, the interaction between the host plant *Silene latifolia* and its pollinator/seed predator partner *Hadena bicruris* is also facultative and even considered a basic state of nursery pollination (Bernasconi et al., 2009; Dufaÿ & Anstett, 2003), often being referred to as parasitic due to the extent of seed predation (Giménez‐Benavides, Dötterl, Jürgens, Escudero, & Iriondo, 2007; Kula, Castillo, Dudash, & Fenster, 2014; Prieto‐Benítez et al., 2017; Reynolds et al., 2012). This interaction, however, is just one pair belonging to a complex formed by plants from the Caryophyllaceae family and moths from the *Hadena* genus. Kephart et al. (2006), and later Prieto‐Benítez et al. (2017) reviewed this system and found a total of 21 different *Hadena* species which predated upon flowers and seed capsules of 70 caryophyllaceous plant hosts during the larval stage, interactions ranging from antagonisms to facultative mutualisms.

The cost/benefit analysis of these interactions is never simple, as associations between organisms do not evolve in isolation, but rather within a complex ecological context, where third parties—such as copollinators, exploiters, predators, or parasites—may play an important role modifying the plant–insect interaction (Bronstein, Wilson, & Morris, 2003; Gomulkiewicz, Nuismer, & Thompson, 2003; Harrison, 2014; Holland & Fleming, 2002; Schatz, Magali, Rakhi, Borges, & Hossaert‐McKey, 2006; Scopece, Campese, Duffy, & Cozzolino, 2018). This means that the net outcome of the interaction may change from a mutualism to an antagonism or commensalism in a reversible fashion depending on the specific environment in which the interaction occurs (Bronstein, 1994; Bronstein, Alarcon, & Geber, 2006; Dufaÿ & Anstett, 2003; Pellmyr, 1989; Pellmyr et al., 1996; Thompson & Cunningham, 2002; Thompson & Fernandez, 2006; Thompson & Pellmyr, 1992; Westerbergh, 2004; Westerbergh & Westerbergh, 2001). Taking this into account, these interactions should not be described as being either fully antagonistic or fully mutualistic, but be placed somewhere along a gradient between antagonism and mutualism (Bronstein, 1994). Nevertheless, until recent, known cases were often described in the extreme categories rather than along a continuum (Anstett et al., 1996; Janzen, 1979; Pellmyr, 1989; Pettersson, 1991a, 1991b; Thompson & Pellmyr, 1992). To further our understanding, it is thus important to elucidate under which ecological circumstances these systems may shift along the aforementioned gradient. From the plants’ perspective, any factor that enhances the plants’ reproductive success, such as interactions with natural enemies of the seed predators that would interfere with herbivore consumption, could be a first step in that shift toward a mutualism. As mentioned above, the *S. latifolia*–*H. bicruris* system is not a specialized mutual system: Copollinators are present in the system and *Hadena* lacks active pollination, meaning there is an absence of any specific morphological structures and behaviors to assure the pollination process (Pellmyr, 1997). This lower degree of specialization makes the system less robust, and therefore likely to shift along the gradient over short periods of time (Bronstein, 1994; Kephart et al., 2006; Thompson & Cunningham, 2002; Westerbergh, 2004). This shift is dependent on the specific context in which the system occurs, and the third parties involved, such as copollinators, parasitoids, or other natural enemies. The question therefore arises: how exactly may these third parties influence the outcome of the interaction?

To answer this question, Bronstein et al. (2003) developed general models to explain how antagonists (such as predators and parasites of the pollinators) could affect population dynamics and evolution of the mutualist partners. Some of the outcomes suggested antagonist species could alter population sizes of the mutualists in such a way that they could stabilize the dynamics of the interaction. In an empirical case study, though not of a nursery pollination system, van Loon, Boer, and Dicke (2000) found that parasitization of the herbivore *Pieris rapae* significantly reduced seed loss of its host plant *Arabidopsis thaliana*, suggesting that parasitism of herbivores potentially increased plant fitness. In that line, a study by Nunes et al. (2018) described a new nursery pollination system formed by a weevil and its orchid host plant, in which parasitoid wasps mediated the outcome of the interaction by killing the weevil larvae and therefore changing the cost/benefit ratio of the partnership. Moreover, very recently Stucchi, Giménez‐Benavides, and Galeano (2019) developed a population dynamics model demonstrating how the *Silene–Hadena* system might be more stable in the presence of parasitoids. Therefore, parasitic wasps of the pollinator *H. bicruris* have the potential to substantially alter the balance of the pollinator/predator and host plant interaction (Harrison, 2014; Holland & Fleming, 2002; Schatz et al., 2006).

On the other hand, *S. latifolia* produces large numbers of seeds per capsule (several hundred, Brantjes, 1976b; Jolivet & Bernasconi, 2007; Young, 2002), and therefore, it is unlikely that the plant is seed limited. As mentioned, *S. latifolia* depends on moth pollination and has gravity seed dispersal, therefore it will have a short dispersal range (Barluenga et al., 2011). According to a study by Peroni and Armstrong (2001), where they estimated seed density and dispersion by monitoring seedling emergence from soil cores, *S. latifolia* seeds follow a clumped dispersion pattern, with very high estimated densities per m^2^. It is known that certain plant species can have negative density‐dependent recruitment, meaning that seedling survival decreases with local conspecific seed density (Jansen, Visser, Wright, Rutten, & Muller‐Landau, 2014; Sheffer, Canham, Kigel, & Perevolotsky, 2013). Yoda, Kira, Ogawa, and Hozumi (1963) identified self‐thinning as one of the main effects of intraspecific competition in plants. Waser, Campbell, Price, and Brody (2010) concluded that the probability of survival until adulthood, and the total number of flowers produced were density dependent. Whereas many studies use fecundity as a direct measure for fitness, this relationship might not always be so straight forward. This was very well shown by Campbell, Brody, Price, Waser, and Aldridge (2017) in an experiment with *Ipomopsis aggregata* plants, where offspring recruitment and reproduction were higher for seeds from low‐fecundity parents due to density‐dependent effects. Therefore, in an intraspecific competition scenario for *S. latifolia* plants, *H. bicruris* larvae might be less detrimental as expected to the plant's reproductive success and fitness, as it could be reducing part of this intraspecific competition by predating on a portion of the seeds.

In this paper, we specifically address two research questions: can a natural enemy impact the level of seed consumption by the seed predator, and if so, what are the consequences at the level of individual plant fitness. We used the *S. latifolia*–*H. bicruris* interaction as a model system, and the ectoparasitoid *Bracon variator* as a natural enemy. We investigated seed output, germination, survival to adulthood, and lifetime flower production as proxies for individual plant fitness with a series of laboratory and greenhouse experiments.

## 2 MATERIALS AND METHODS

### 2.1 The model system: the *Silene latifolia–Hadena bicruris–Bracon variator* interaction

The host plant: The White Campion *Silene latifolia* (Caryophyllaceae) is a short‐lived perennial weed that exists in natural metapopulations and is normally found in open disturbed habitats such as field margins, roadsides, or grazing fields (Elzinga, Harvey, & Biere, 2003; Elzinga, Nouhuys, Leeuwen, & Biere, 2007; Elzinga, Zwakhals, Harvey, & Biere, 2007). Plants of the *Silene* group are dioecious, and although dioecy is widespread in plants, this characteristic makes *Silene* plants quite unique within the nursery pollination systems, as they are the only dioecious plants in which the sexual function of the tissues eaten by the larvae of the pollinator is the female one (Dufaÿ & Anstett, 2003). In all other systems consisting of dioecious plants reviewed by Dufaÿ and Anstett (2003), the larvae attacked the tissues with male sexual function. When pollinator larvae develop at the expense of the male structures, there is low or no cost to the plant, as its pollen has already been dispersed and the reproductive episode is over (Dufaÿ & Anstett, 2003). However, in the case of *Silene* plants, pollinator larvae will feed on the fruit and seeds, thereby imposing high costs to the plant (Dufaÿ & Anstett, 2003). These high costs and sex‐specific fitness consequences due to the attack upon the female plants, makes *Silene* the perfect model plant to study the early stages of evolution in nursery pollination mutualisms (Westerbergh, 2004).

**Figure 1:**
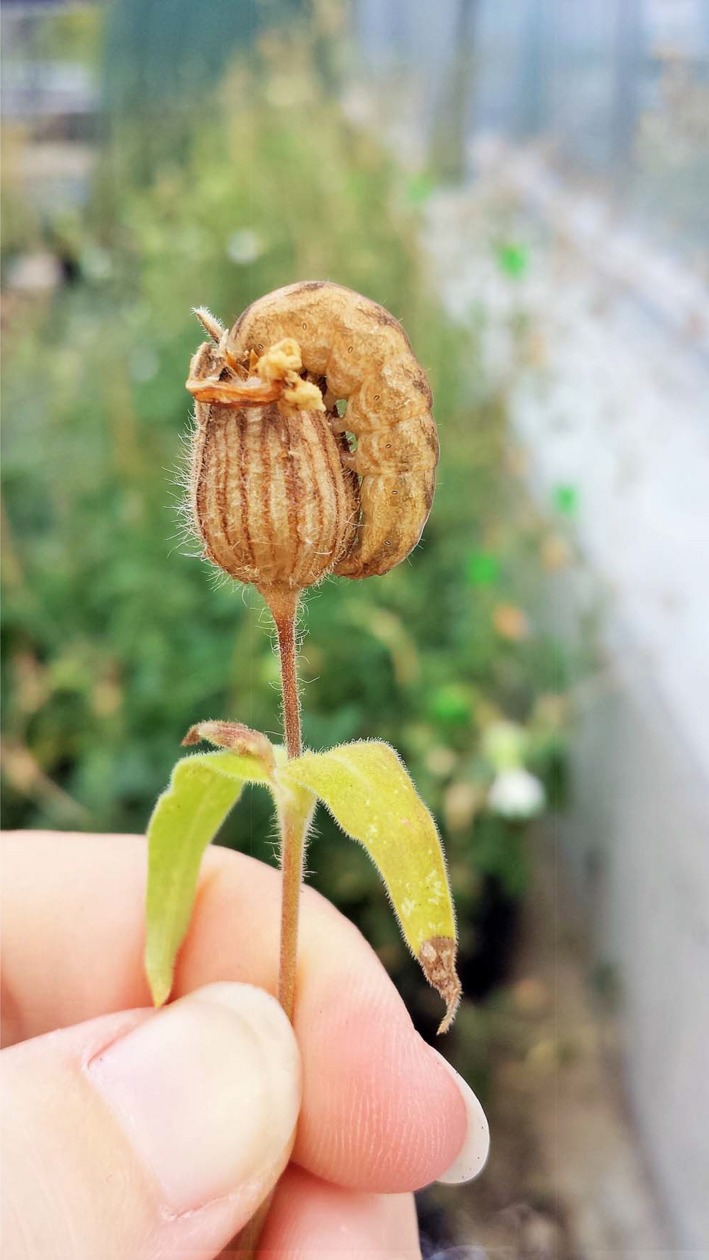
*Hadena bicruris* larva at a late developmental stage feeding on a *Silene latifolia* secondary seed capsule

The pollinator/herbivore: Adult Lychnis Moths, *Hadena bicruris* (Lepidoptera; Noctuidae), are the main pollinator of *S. latifolia* plants, but this species is also their most important seed predator (Brantjes, 1976b; Elzinga, Turin, Damme, & Biere, 2005; Kephart et al., 2006). Both males and females are active at night and fly from male to female plants, feeding on nectar and passively pollinating the flowers. Female moths also oviposit a single egg on female flowers and use cues to avoid superparasitism by leaving a volatile oviposition deterrent which indicates the flower has already been parasitized by a conspecific (Brantjes, 1976a, 1976b; Roitberg & Prokopy, 1987). After egg eclosion, the young larva feeds on the developing seeds inside the seed capsule where it had hatched (Elzinga et al., 2005). Once it has grown to a late developmental stage and has consumed all of the seeds in the primary capsule the larva moves to secondary capsules for feeding (Figure 1), destroying up to five other capsules on the same plant (Brantjes, 1976a; Elzinga et al., 2005). *Hadena bicruris* is widely spread in *S. latifolia* populations, where it was found in over 90% of plant populations in Western Europe, although at varying densities (Elzinga et al., 2005). The degree of seed capsule destruction varies greatly, with an average of 50% of all fruits being destroyed (Biere & Honders, 1996; Elzinga et al., 2005); thus, periods with high seed destruction have a major impact on plant fitness (Biere & Honders, 1996). The interaction is usually described as an antagonistic one, with *H. bicruris* parasitizing *S. latifolia* (Brantjes, 1976b); however, the degree to which it may be antagonistic may vary from year to year.

The natural enemy: The parasitoid wasp *Bracon variator* (Hymenoptera; Braconidae) has been found to attack *H. bicruris*, although it occurs at low incidence in the field (Elzinga, Zwakhals, et al., 2007). As an idiobiont ectoparasitoid, it oviposits on the surface of the host, immobilizing it by injecting a paralyzing venom which also prevents any further development of the host (Askew & Shaw, 1986). This species attacks hosts in their L3‐L5 instars, and by arresting host development prevents further seed consumption by the herbivore (Elzinga, Zwakhals, et al., 2007). Therefore, *B. variator* can cause a decrease in predation of *S. latifolia* seeds by parasitizing *H. bicruris* larvae, potentially reducing the costs of the interaction for the plant.

### 2.2 Experiment 1: the role of *B. variator* as a natural enemy

#### 2.2.1 Rearing of individuals

*S. latifolia* plants were reared in the cold‐frame greenhouse facilities of the experimental garden of the University of Bremen over the summers of 2017 and 2018 from seeds collected from natural field populations in the municipality of Ottersberg (53.1102, 9.1512; Lower Saxony), close to Bremen. We used large seedling trays (8 × 12 cells; ø6 cm) for sowed seeds and transplanted the seedlings to 6 cm ø pots at the six‐leaf stage. Once the basal rosette was formed, plants were transplanted to their final pots (11 × 11 × 10 cm). Temperature and light were not controlled for in the cold‐frame and were dependent on environmental conditions. Plants were watered as needed. Plants were divided into two groups: experimental plants and plants for the rearing of insects. To make sure that experimental plants and flowers had no previous contact with pollinators, once they started blooming both male and female plants were kept in 1m^3^ net tents (Nature^®^). Laboratory populations of *H. bicruris* and *B. variator* were established in both years from individuals collected from the field at the beginning of the season and replenished with individuals found in the greenhouse plants used for rearing. All collected individuals were kept inside climate cabinets at 23°C with a 16L:8D light regime. Parasitoid clutches collected in the field were kept separately in small plastic vials until all offspring hatched. Adult parasitoids were kept in population boxes where they could mate and were fed on drops of honey and water. Newly hatched and young instar *H. bicruris* larvae were fed freshly pollinated *S. latifolia* capsules with tender developing seeds to increase survival rate, while late instar larvae were fed with an artificial diet, prepared according to Elzinga, Biere, and Harvey (2002), which was refreshed on alternate days. All larvae were kept separately in small plastic vials to avoid cannibalism and once pupated they were moved to a pupation box, which was checked daily for emerging adults. Newly hatched *H. bicruris* adults were sexed and moved to population boxes (34.5 × 22 × 30 cm), sorted by age and fed a honey–water solution.

#### 2.2.2 Reduction of seed predation by parasitoid attack

We measured the impact of the parasitoid wasp *B. variator* on larval seed predation and hypothesized that the parasitization of *H. bicruris* larvae by *B. variator* would reduce seed loss in *S. latifolia* plants.

The experiment to test seed production of plants (a) “without herbivore attack,” (b) “with herbivore attack,” and (c) “with herbivore attack plus parasitoids” was carried out under controlled laboratory conditions by employing collapsible insect rearing cages (60 × 60 × 90 cm, Aerarium^®^). Each cage contained a single female *S. latifolia* plant with a minimum of six open flowers, and a male *S. latifolia* plant with a minimum of 10 open flowers to ensure sufficient pollen for pollination. All plants used belonged to the group of experimental plants reared in the cold‐frame greenhouse. A 4–6 days old mated female, previously starved for 24 hr, was added to the cage in the evening and left to feed and pollinate the flowers overnight. The next morning the moths were removed. Each replicate consisted of three treatments: (a) “control” treatment (negative control): After pollination by the female moth, all eggs were removed from the plant to avoid infestation by *H. bicruris*; (b) “herbivore” treatment: *S. latifolia* plants pollinated and parasitized by a single *H. bicruris* larva which fed undisturbed (to ensure this, all visible eggs except one visibly fertilized egg that was haphazardly chosen were removed from the plant, and in case this method failed and more than one larva was detected later the replicate was discarded and repeated); and (c) “herbivore + parasitoid” treatment: *S. latifolia* plants pollinated and parasitized by a single *H. bicruris* larva; once the larva emerged from the primary capsule to move to secondary capsules, a mated and experienced *B. variator* female was released inside the cage until parasitism of the larva. The end of each replicate was marked as the moment when the larva from the herbivore treatment had either pupated or been paralyzed by the parasitoid. At this point, all capsules, damaged or undamaged, were counted, gathered, and stored individually and per plant. All seeds were later counted, and data on the number of seeds per capsules and per plant were collected. Each capsule and all its seeds (without plant tissue) were also weighed on a precision scale (Quintix^®^, Sartorius Lab Instruments) to collect data on average seed weight per capsule.

#### 2.2.3 Early germination

Rapid germination is a quality of many ruderal plants and especially of those growing near arable fields (Grime, 1977). It benefits plants as they can start using resources to outcompete other individuals, therefore timing and speed of germination can be crucial for a successful seedling establishment (Gioria & Pyšek, 2017). In order to test if there is any qualitative change in the seeds due to attack by *H. bicruris*, we carried out an early germination test, with the seeds obtained from the previous experiment. We hypothesized that seeds coming from unattacked plants would have a higher quality and therefore earlier germination, while seeds from damaged capsules would have a lower quality than those coming from undamaged capsules. The setup included three different treatments: (a) seeds from pollinated plants without herbivore attack, (b) seeds from undamaged capsules from plants with herbivore attack (with and without parasitoid), and (c) seeds from damaged capsules from plants with herbivore attack (with and without parasitoid). We used large seedling trays (8 × 12 cells; ø6 cm) and sowed each seed in one separate cell, randomly allocating the treatments. Seeds were watered every day and kept inside the greenhouse facilities of the experimental garden of the University of Bremen. After 7 days, the trays were checked for germination (recorded as 0 if the seed had not germinated or 1 if the seed had germinated). In our experience, *S. latifolia* in greenhouse conditions would typically start germinating in 3–5 days, and therefore, we consider a week enough time to see differences in the early germination of seeds from different treatments, as proxy for seed quality.

#### 2.2.4 Statistical analyses

**Figure 2:**
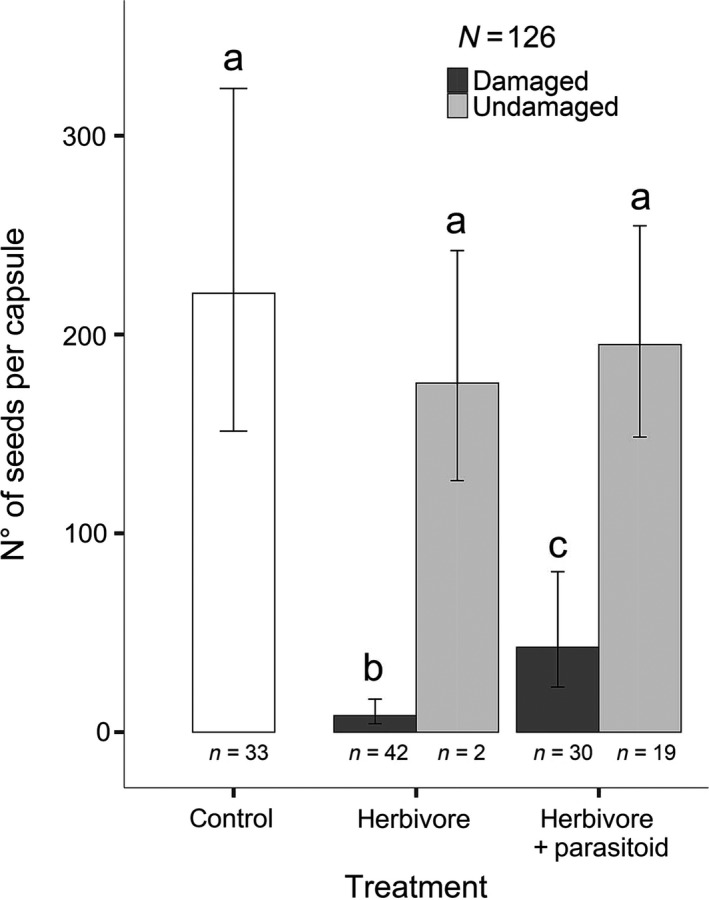
Seed output per capsule in *Silene latifolia* plants under different treatments: “control” (negative control, no presence of the larvae and hence no predation), “herbivore” (larva is present and allowed to feed freely on seed capsules) and “herbivore + parasitoid” treatment (the predating larva is attacked by the parasitoid *Bracon variator*). Undamaged capsules (light gray filled bars) escaped predation by the larva of *Hadena bicruris*, while damaged capsules (dark gray filled bars) were predated upon by the larva *H. bicruris.* The bars represent model estimates and confidence intervals. (GEE Model with a poisson error distribution and log‐link function, id = plant, corstr = exchangeable; *Χ* ^2^ *_df_*_=4;_ *_n_*_=126_ = 122.67; *p*‐value = 2.20e−16)

**Figure 3:**
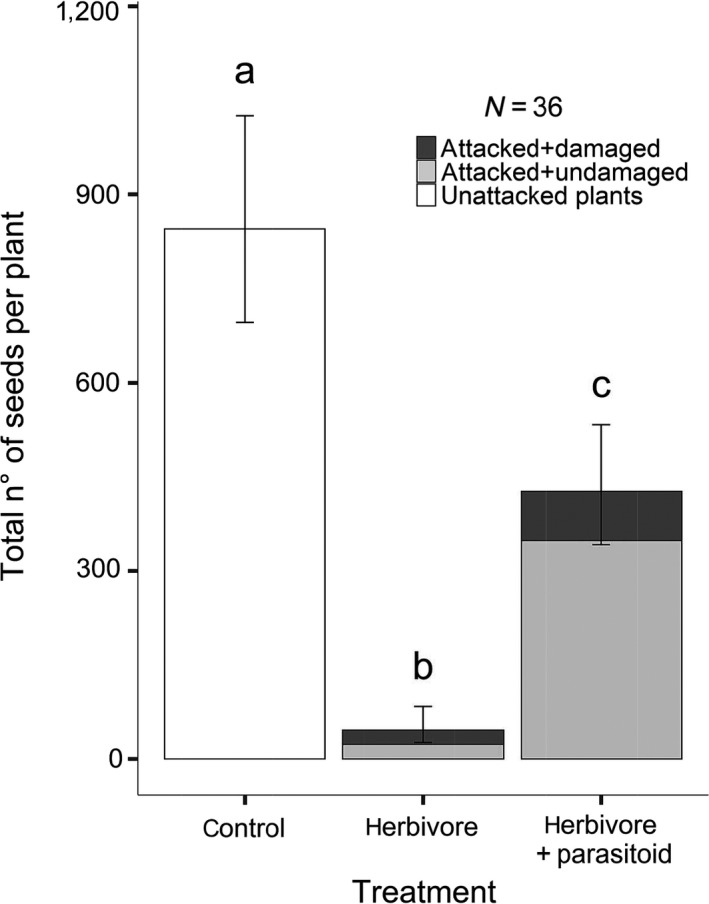
Total seed output per plant for *S. latifolia* plants under different treatments: “control” (negative control, no presence of the larvae and hence no predation), “herbivore” (larva is present and allowed to feed freely on the plant) and “herbivore + parasitoid” (treatment, the predating larva is attacked by the parasitoid *B. variator*). Undamaged capsules (light gray filled bars) escaped predation by the larva of *Hadena bicruris*, while damaged capsules (dark gray filled bars) were predated upon by the larva *H. bicruris.* The bars represent model estimates and confidence intervals. (GLM with a poisson error distribution with a correction for overdispersion and log‐link function; *F*_[2,33]_ = 21.51; *p*‐value = 1.48e−06; McFadden's *R^2^* = 57,43%)

**Figure 4:**
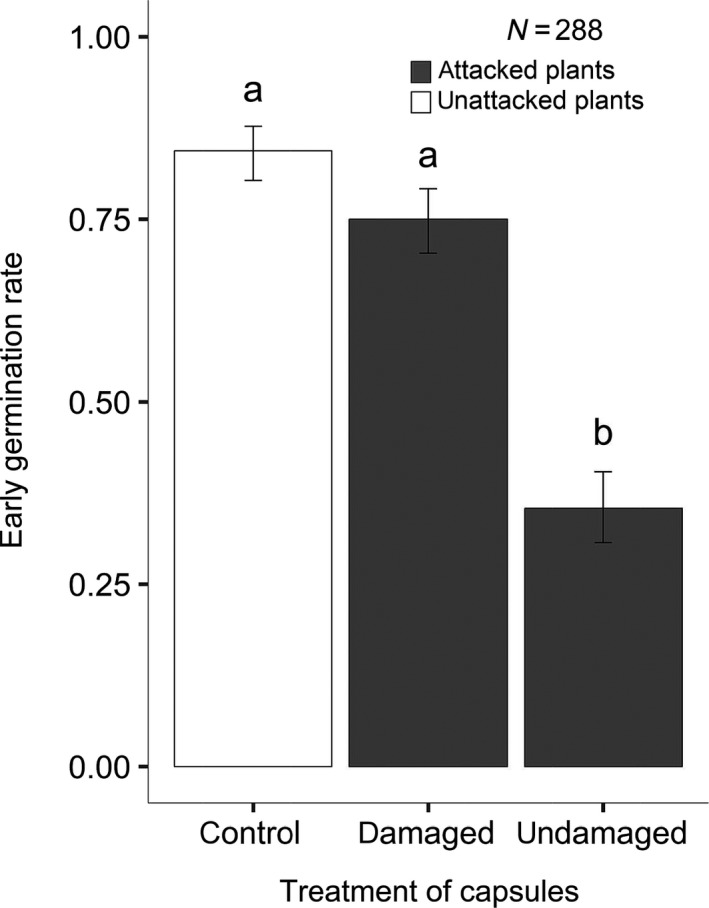
Early germination for *S. latifolia* seeds under different treatments: “control” (seeds from capsules from pollinated plants without herbivore attack), “damaged” (seeds from damaged capsules from plants with herbivore attack), and “undamaged” (seeds from undamaged capsules from plants with herbivore attack). The bars represent model estimates and confidence intervals. (GLM with a binomial error distribution and logit‐link function; *Χ* ^2^ *_df_*_=2;_ *_n_*_=288_ = 57.20; *p*‐value = 3.80e−13; McFadden's *R* ^2^ = 15.33%)

All statistical analyses were performed using “R” (Version 3.5.3) for statistical computing (R Core Team, 2019) and the interface “RStudio” (RStudio Team, 2016). Seed output per capsule was analyzed taking into account the treatment of the plant (“control,” “herbivore,” and “herbivore + parasitoid” treatments) and whether the capsules were damaged (attacked by *Hadena*) or not. The combination of both factors gives five categories: (a) “control” (always undamaged as there is no presence of larvae), (b) “herbivore + undamaged” (capsules produced by a plant in the respective treatment that were not attacked by the larva of *H. bicruris*), (c) “herbivore + damaged” (capsules attacked by the larva *H. bicruris*), (d) “herbivore + parasitoid+undamaged” (capsules produced by a plant in the respective treatment that escaped larval attack from *H. bicruris*) and (e) “herbivore + parasitoid + damaged” (capsules attacked by the larva *H. bicruris*, which eventually was parasitized by *B. variator*). To analyze seed output per capsule and mean seed weight, we used Generalized Estimating Equation (GEE, Hardin & Hilbe, 2002) models, function “geeglm” from the package “geepack” (Højsgaard, Halekoh, & Yan, 2006; Yan, 2002; Yan & Fine, 2004). By using GEE with “id = plant” and correlation structure “exchangeable” (for seed output) and “ar1” (for mean seed weight), we corrected for data correlation in repeated measurements, that is, the measurement of several capsules per plant. We used poisson error distribution (for count data) with a log‐link function. In addition, we performed contrast tests following the close test principal (Bretz, Hothorn, & Westfall, 2010) with the function “esticon” from the package “doBy” (Højsgaard & Halekoh, 2018). Generalized linear models (GLM, Nelder & Wedderburn, 1972) were applied to analyze total seed output per plant using the package “car” (Fox & Weisberg, 2011) and a poisson error distribution with correction for overdispersed data and a log‐link function. To analyze the proportion of undamaged capsules in different treatments and early germination rate, we also used GLMs, this time using binomial error distributions with logit‐link funtion. In either cases, there was a need to correct for multiplicity as treatment consisted only of three groups. Therefore, contrast tests were applied between pairs of categories using the package “contrast” (Kuhn, S. contributions from Weston, J. Wing, J. Forester, & T. Thaler., 2016). Finally, package “ggplot2” (Wickman, 2016) was used to create the bar graphs in Figures 2, 3, 4 and Figure S1. Package “emmeans” (Lenth, 2019) was used to calculate the confidence intervals represented in Figure 2 and Figure S2.

### 2.3 Experiment 2: consequences for plant fitness

We measured the impact of initial seed density on the number of germinating seeds, seedling to adult plant recruitment and flower anthesis in adult female and male *S. latifolia* plants (measured as the total number of open flowers in a lifetime) as a proxy for individual plant fitness. We hypothesized that at higher densities, germination, survival, and flower production would decrease due to intraspecific competition.

We created artificial seed densities by extrapolating the estimated mean of 535.29 seedlings/m^2^ (Peroni & Armstrong, 2001) to the dimension of our pots (11 × 11 × 10 cm), which gave us a density of 6.48 seedlings per pot. Taking this information into account and looking at our results from the previous experiment of total seed production, we decided to create seed densities of 1, 2, 3, 4, 5, 6, 7, 8, 9, 10, 20, 40, 80, and 150 seeds per pot. All seeds used were randomly selected from a pool of seeds collected at the field sites described in the methods section. This was repeated for five simultaneous replicates. The experiment was setup in the cold‐frame greenhouse facilities of the experimental garden of the University of Bremen, where temperature and light were not controlled for but rather dependent on environmental conditions. Pots were watered as needed. After 10 days, the number of seedlings (germinated seeds) was counted.

Plants were reared until the adult stage. Stems were given individual codes, as at high densities it was hard to distinguish between plants, and their sex was recorded. Plants were checked every day to collect data on the number of new open flowers produced per stem. After being counted, open flowers were picked from the plant. This was done until all stems died. At this point, the experiment was concluded by removing plants from their pots to identify the origin of the stems, and surviving plants from each pot were counted to obtain data for seedling to adult plant recruitment.

#### 2.3.1 Statistical analyses

All statistical analyses were performed using “R” (Version 3.5.3) for statistical computing (R Core Team, 2019) and the interface “RStudio” (RStudio Team, 2016). Graphical analysis of the proportion of surviving plants as a function of density suggested the responses were density dependent so we fitted nonlinear regression models (Bates & Watts, 1988) with the function “nls” (nonlinear least squares) in the native “stats” package, and we used the “nlme” (Pinheiro, Bates, DebRoy, Sarkar, & Team, 2018) package to check the residuals. Models for germination and survival probability were fitted following a logarithmic equation (*y* = *a* + b*log(*x*)) where *x* is the initial seed density and *a* and *b* are the regression coefficients. The response variable total flower anthesis per plant was log‐transformed to achieve a normal distribution, and it was analyzed with an additive Linear Mixed Model (LMM, Zuur et al. 2009) as a function of density, sex, and number of stems as a random factor, using package “lme4” (Bates, Maechler, Bolker, & Walker, 2015), and package “MumIn” (Barton, 2009) to obtain an *R^2^* value for the same model.

## 3 RESULTS

### 3.1 Seed output

We found significant differences in the seed output of capsules from different categories (Figure 2; *Χ* ^2^ *_df_*_=4;_ *_n_*_=126_ = 122.67; *p*‐value = 2.20e−16; GEE poisson distribution with log‐link function, id = plant, corstr = exchangeable). The contrast test between all pairs of categories (Table 1) showed that undamaged capsules from the three treatments (“control,” “herbivore + undamaged,” and “herbivore + parasitoid + undamaged”) do not significantly differ in their seed outputs. However, the seed output of undamaged capsules was significantly different to the seed output of damaged capsules from both treatments (“herbivore + damaged” and “herbivore + parasitoid+damaged,” respectively), the latter two also being significantly different from each other. While capsules from the herbivore treatment that were attacked by the larvae barely produced a seed output of a few surviving seeds, capsules attacked by *H. bicruris* and later parasitized by *B. variator* had a significantly higher seed output yet lower than the seed output from undamaged seed capsules (Figure 2). Moreover, the “herbivore + parasitoid” treatment produced a significantly higher proportion of undamaged capsules compared to the “herbivore treatment” (*Χ* ^2^ *_df_*_=1;_ *_n_*_=28_ = 19.245; *p‐*value = 1.10e−05; see Figure S1). On the other hand, mean weight of seeds in *S. latifolia* capsules under different treatments was not significantly different (*Χ* ^2^ *_df_*_=4;_ *_n_*_=85_ = 3.76; *p*‐value = .44; see Figure S2).

**Table 1:** Contrast tests following the close test principal (Bretz et al., 2010) for seed output per capsule between all pairs of categories

*p*‐Values	Herbivore + parasitoid + undamaged	Herbivore + parasitoid + damaged	Herbivore + undamaged	Herbivore + damaged
Herbivore + parasitoid+damaged	4.63e−05***	–	–	–
Herbivore + undamaged	n.s	0.0001***	–	–
Herbivore + damaged	1.11e−16***	0.0006***	<0.0001***	–
Control	n.s	1.36e−05***	n.s	2.22e−16***

Significant differences between pairs are shown in bold (*p < .05; **p < .01; ***p < .001).

Seed output per plant was analyzed counting the total number of seeds contained in all capsules from each plant. This was done for all three treatments (“control,” “herbivore,” and “herbivore + parasitoid”). Treatment has a significant effect on the total seed output per plant (Figure 3; *F*_[2,33]_ = 21.51; *p*‐value = 1.48e−06; McFadden's *R* ^2^ = 57,43%; GLM poisson distribution with log‐link function). The “control” treatment had a significantly higher (*p*‐value = 0.029) total seed output per plant compared with the “herbivore + parasitoid” treatment, which in turn had a significantly higher (*p*‐value = 0.0013) total seed output than the “herbivore” treatment with the lowest total seed output per plant. The same pattern was observed when we analyzed the total number of seeds as a function of treatment (*F*_[2,32]_ = 24.82; *p*‐value = 3.1e−07) and total number of capsules produced per plant (*F*_[1,32]_ = 8.46; *p*‐value = 0.0066) with an additive model (McFadden's *R* ^2^ = 67,60%; see Figure S3).

### 3.2 Early germination

Overall, we found significant differences in early germination between seeds coming from different treatments (Figure 4; *Χ* ^2^ *_df_*_=2;_ *_n_*_=_ = 57.20; *p*‐value = 3.80e−13; GLM binomial distribution with logit‐link function). Seeds from capsules from pollinated plants without herbivore attack and seeds from damaged capsules from plants with herbivore attack both had high early germination (84.4% and 75%, respectively), and the contrast test showed no significant difference between these two treatments (*p*‐value = 0.11), which was against our initial expectations. Seeds from undamaged capsules from plants with herbivore attack had a low early germination of 35.4%, and the contrast test confirmed this result was significantly lower compared with the previous treatments (*p*‐value < .0001). However, the variance explained through our model is very low (McFadden's *R* ^2^ = 15.33%), and therefore, it is very likely that there are other processes involved that we are not aware of.

### 3.3 Density‐dependent effects

**Figure 5:**
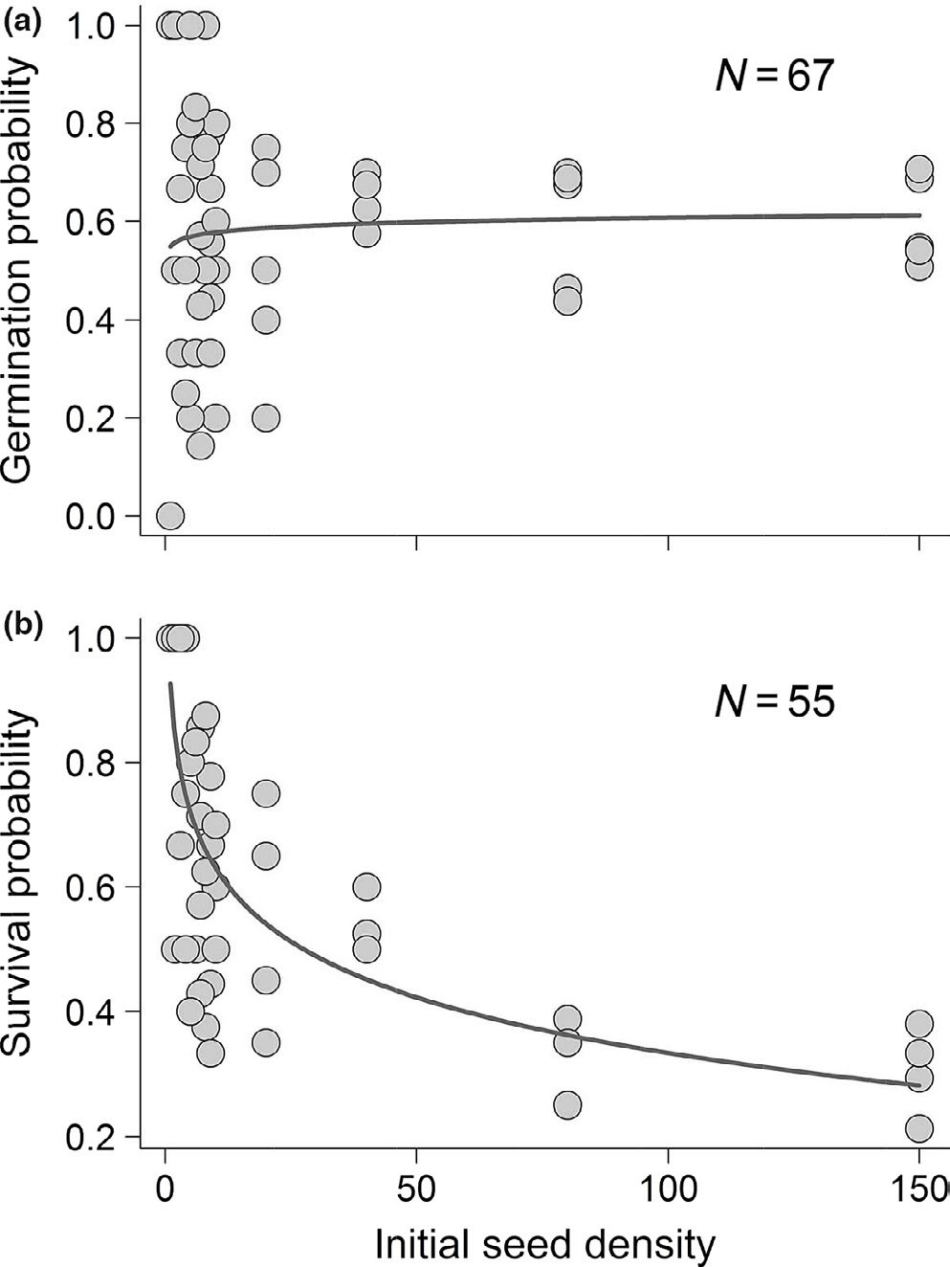
Germination (a) and survival (b) probability of *S. latifolia* plants as a function of initial seed density, represented by the best fitting lines (NLS Models, fitted to a logarithmic equation (*y* = *a *+ b*log(*x*)), starting values: *a* = 0.1, *b* = 0.1; (a) coeff.a = 0.55, *SE* = 0.057, *t*‐value = 9.60, *p*‐value = 2.84e−14; coeff.b = 0.013, *SE* = 0.022, *t*‐value = 0.58, *p*‐value = 0.57; *r^2^ = *0.48%; (b) coeff.a = 0.93, *SE* = 0.044, *t*‐value = 21.14, *p*‐value < 2e−16; coeff.b = −0.13, *SE* = 0.017, *t*‐value = −7.71, *p*‐value = 3.25e−10; *r^2^ = *52.87%)

**Figure 6:**
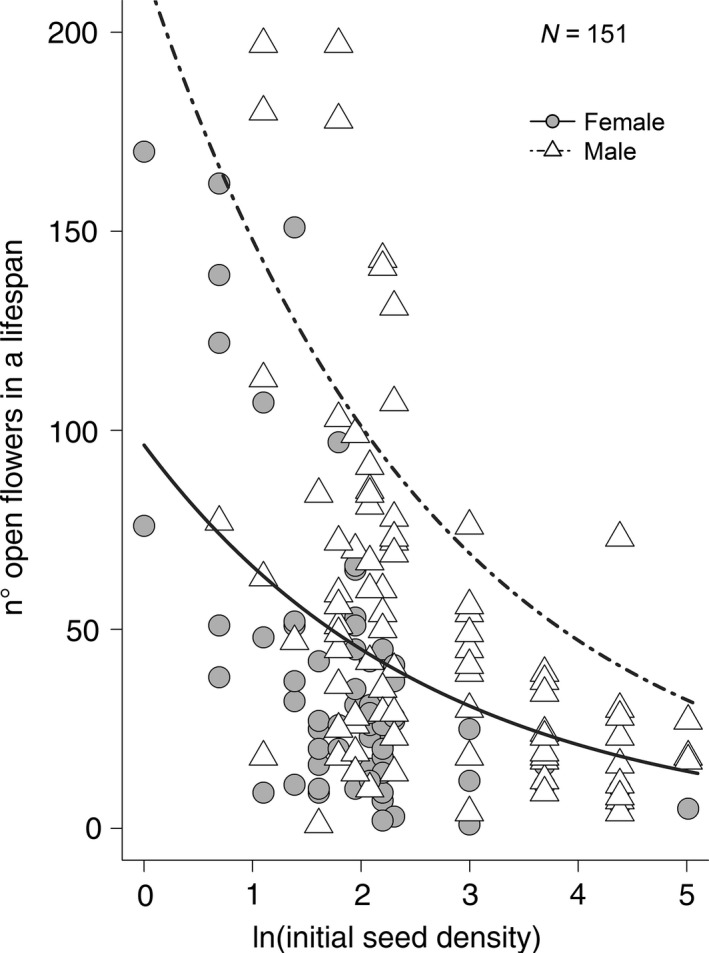
Total flower anthesis per plant in *Silene latifolia* as a function of initial seed density (*Χ* ^2^ *_df_*_=1;_ *_n_*_=151_ = 30.10; *p*‐value = 4.20e−08) and sex (*Χ* ^2^ *_df_*_=1;_ *_n_*_=151_ = 35.50; *p*‐value = 2.60e−09), represented by the best fitting lines (LMM with number of stems as random term; model estimates have been backtransformed to fit the original data; females: *y*_1_ = exp(−0.38*x + 4.57); males: *y*_2_ = exp(−0.38*x + 5.38); conditional *R* ^2^ = 48.18%)

We found that the germination probability did not follow a density‐dependent response (Figure 5a; coeff.a = 0.55, *SE* = 0.057, *t*‐value = 9.60, *p*‐value = 2.84e−14; coeff.b = 0.013, *SE* = 0.022, *t*‐value = 0.58, *p*‐value = 0.57; NLS Model). Yet, survival probability did follow a density‐dependent logarithmic response to initial seed density (Figure 5b; coeff.a = 0.93, *SE* = 0.044, *t*‐value = 21.14, *p*‐value < 2e−16; coeff.b = −0.13, *SE* = 0.017, *t*‐value = −7.71, *p*‐value = 3.25e−10; NLS Model). As initial seed density increases, there is a strong decrease in the proportion of plants that survive to adulthood. In addition, the analysis of the total flower anthesis per plant showed significant effects of both initial seed density (*Χ* ^2^ *_df_*_=1;_ *_n_*_=151_ = 30.10; *p*‐value = 4.20e−08) and sex (*Χ* ^2^ *_df_*_=1;_ *_n_*_=151_ = 35.50; *p*‐value = 2.60e−09; Figure 6; LMM with number of stems as random term; conditional *R^2^* = 48.18%). We can see a strong negative effect of initial seed density on the total number of flowers produced starting at low densities, with an overall lower production of flowers in female plants (*y*_1_ = exp(−0.38*x + 4.57)) than male plants (*y*_2_ = exp(−0.38*x + 5.38)) (model estimates have been backtransformed to calculate the best fitting lines for the original data, with a poisson distributed response variable).

## 4 DISCUSSION

This study aims to determine whether the parasitoid *B. variator* has an impact on the level of seed consumption by the seed predator *H. bicruris,* and thus, whether it modifies the interaction between the plant and herbivore along the mutualism–antagonism gradient. To this end, we tested how parasitoid action translates into possible consequences in individual plant fitness*.* At first, our results support our first hypothesis, suggesting that *B. variator* can indeed decrease the level of seed predation by the larvae of *H. bicruris,* therefore reducing the costs of the interaction for the host plant partner *S. latifolia*. However, to which extent this increase in seed output is translated into an increase in plant fitness is something that needs to be discussed.

Under our experimental conditions, the parasitism of *H. bicruris* by *B. variator* resulted in an increase in seed output in *S. latifolia* at both the capsule and plant level. The fact that there are no significant differences between the seed outputs of undamaged capsules suggests that infested and uninfested plants allocate their resources per capsule equally. We conclude that the increase in seed output shown by damaged capsules and plants where the natural enemy was present is a direct result of the parasitism by the parasitoid *B. variator* and not due to a differential allocation of resources in plants assigned to different treatments.

Nursery pollination systems require a balance between the costs and the benefits of the interaction for both partners to achieve a positive outcome, which means that there must be mechanisms which prevent overexploitation by either mutualistic partner or which ensure survival of future generations. These mechanisms are varied, ranging from selective abortion of infested fruits, cannibalism, or changes in phenology, to the presence of third parties (Brantjes, 1976a; Burkhardt, Ridenhour, Delph, & Bernasconi, 2012; Holland & DeAngelis, 2006; Reynolds et al., 2012; Stucchi et al., 2019; Wright & Meagher, 2003), or seed dispersal in space and time (e.g., seed dormancy; Fenner & Thompson, 2005). Seed dormancy, defined as failure of an intact viable seed to complete immediate germination under favorable conditions (Bewley, 1997), may lead to variation in seed dispersal in time. The function of dormancy is crucial as it prevents germination when the probability of survival of the seedling is low (Fenner & Thompson, 2005). The general mechanisms of seed dormancy are well studied and understood (see reviews by Finch‐Savage & Leubner‐Metzger, 2006; Bentsink & Koornneef, 2008, Nonogaki 2014), while the same cannot be said about the specific mechanisms by which parental plants can alter the dormancy state of seeds. Baskin and Baskin (1998) reviewed the effects of parental plants on seed dormancy, in cases where parent plant detects a particular stimulus and responds to it by altering the level of dormancy (Fenner & Thompson, 2005). Although the mechanisms are not well understood, a recent study by Singh et al. (2017) on *Arabidopsis thaliana* showed that herbivory pressure suffered by the maternal plant can result in the loss of dormancy in its offspring, a process regulated by phytohormones.

Our germination rate results suggest that there might be some maternal effect occurring. It is possible that the presence of the predator can act as a trigger to increase seed dormancy in seeds from capsules that escaped herbivore attack on infested plants. The germination rates in seeds from attacked plants that did suffer predation by the herbivore vary between damaged and undamaged capsules. These differences in germination rates are not due to seed size differences, as average seed weight was not significantly different between treatments of capsules (see Figure S2). It is possible that attacked plants, as a response to the high herbivore pressure they were suffering in their damaged capsules, increase dormancy levels to seeds from undamaged capsules. Although this contradicts the results from Singh et al. (2017), in our view, it could serve as a possible means to escape from herbivory and increase survival probability. Uninfested plants that did not suffer predation by *H. bicruris* had very high early germination, which is in line with this idea. On the other hand, enhancing seed dormancy in damaged capsules which are currently infested with the herbivore might be a waste of resources, given that without parasitoid attack most of these seeds will be consumed by the herbivore as shown by our results in Figure 2.

Previous works have explored the role of third parties in balancing the costs and benefits in nursery pollination systems. Elzinga et al. (2003) studied parasitism of *H. bicruris* larvae by the koinobiont endoparasitoid *Microplitis tristis* and its effect on larval feeding behavior. Parasitism resulted in lower food consumption of the herbivore and the authors suggested this could positively impact *S. latifolia* populations, although it was only tested on larvae feeding on artificial diet. Various other studies suggest that high rates of parasitism of *H. bicruris* in the field could decrease the seed damage caused by the larvae, and in such cases, the benefits obtained through the adult moth pollinators might counteract the costs of seed predation by the offspring (Biere, Elzinga, Honders, & Harvey, 2002). Later Elzinga et al. (2005) and Elzinga, Zwakhals, et al. (2007) dismissed this idea as in the field the highest incidence rates corresponded to koinobiont parasitoid species (such as *M. tristis*), which do not arrest host growth or seed predation post parasitism. However, in this study, we have a different scenario, as *B. variator* is an idiobiont ectoparasitoid commonly found in the field populations we sampled, which does indeed prevent its host larva from developing and feeding any further, and also by following a clear quantitative approach. Crabb and Pellmyr (2006) showed how a braconid parasitoid wasp could affect seed predation of yucca moth offspring, increasing the production of yucca seeds and reducing the costs of pollination. The already mentioned study by Nunes et al. (2018) showed that parasitoids could rescue part of the fruits of the orchid host plant *Dichaea cogniauxiana* from predation by the weevil larvae, changing the cost/benefit ratio of the host plant and pollinator/herbivore interaction to a positive one. As previously mentioned, the *S. latifolia–H. bicruris* system has been referred to as an antagonistic interaction in the literature due to the extent of seed predation caused by *H. bicruris* larvae, which often impose larger costs than those benefits granted through pollination by adult individuals. In line with these studies, our results show that parasitism by *B. variator* could act as a regulator in the *S. latifolia–H. bicruris* system, reducing the costs imposed by larval feeding and controlling pollinator/seed predator populations, therefore possibly acting as a stabilizing mechanism of the interaction across evolutionary time.

However, our density‐dependent experiments show that increasing seed density can lead to a negative impact in adult plant survival and fitness. The probability to germinate was not density dependent and likely more affected by other unknown factors, like dormancy triggered by *Hadena* predation. On the other hand, in our experimental setting, survival probability rapidly decreased at high densities. Although these densities might only be achieved in the field under ideal conditions, it is likely that *S. latifolia* has a negative density‐dependent recruitment. The same effect was observed for total flower anthesis per plant, with a strong decrease in flower production at high densities. This is in line with studies by Lara‐Romero, Cruz, Escribano‐Ávila, García‐Fernández, and Iriondo (2016) on *Silene ciliata,* in which they report self‐thinning in recruits and a lower adult reproductive performance at higher conspecific density, and also agrees with the already mentioned study by Campbell et al. (2017). This means that even when plants survive, they might suffer the effect of higher densities throughout their lifetime, achieving a lower fitness. Inevitably, this leads us to question what benefit the increase in seed output seen in plants due to parasitism by parasitoid *B. variator* provides to individual plant fitness. Clearly, a higher seed output likely means higher seed density in the soil, and the possible negative effects of competition that may accrue from it. At some conditions, *B. variator* might indirectly be causing an increase in density‐related intraspecific competition and therefore diminishing its positive impact on plant fitness, whereas *H. bicruris* could actually be decreasing intraspecific competition by feeding on *S. latifolia* seeds, increasing the chances of the remaining seeds to be successful. In this scenario, the interaction between *S. latifolia* and *H. bicruris* should not be viewed as antagonistic, but rather much more specialized than it has been considered until now, as it would mean that the host plant invests in high amounts of seed production to compensate for the feeding of its mutualistic partner, and therefore lowers the costs of their interaction.

The parasitism of Hadena by *B. variator* might not only affect the seed availability for the next plant generation, it might also impact the pollination level at the next generation, given it reduces the moth populations considerably and given other pollinators would not compensate for this. This would be an intriguing line of thought best addressed with a modelling approach at the tritrophic population level, but this is beyond the scope of this paper.

Ever since it was demonstrated three decades ago that plants emitted volatile compounds as a response to several forms of herbivory attack (Dicke & Sabelis, 1988; Dicke, Sabelis, Takabayashi, Bruin, & Posthumus, 1990; van Loon et al., 2000; Steidle, Fischer, & Gantert, 2005; Turlings, Tumlinson, & Lewis, 1990) or even oviposition (Hilker & Meiners, 2006), there has been a standing discussion whether plants “crying for help” to attract the natural enemies of their herbivores may actually be beneficial for plant fitness (Dicke & Baldwin, 2010; Heil, 2014; Kessler & Heil, 2011). Only a few studies have provided evidence for a net increase in plant fitness as a direct result of natural enemies attacking their herbivores (Cuny, Gendry, Hernandez‐Cumplido, & Benrey, 2018; Gols et al., 2015; Hoballah & Turlings, 2001; van Loon et al., 2000; Schuman, Barthel, & Baldwin, 2012). More interestingly, recent studies have also shown that in certain cases, natural enemies may confer a negative effect on plant fitness (Smallegange, Loon, Blatt, Harvey, & Dicke, 2008; Xi, Eisenhauer, & Sun, 2015). Smallegange et al. (2008) studied the effect of a koinobiont endoparasitoid on plant fitness and found out that unparasitized caterpillars and caterpillars with high load of parasitoid larvae consumed more flowers than caterpillars with single parasitoid broods, and as a result, there was a decrease in seed production. Xi et al. (2015) concluded that parasitism by a koinobiont endoparasitoid increased seed damage caused by the seed predating larvae of a species of tephritid flies. Our study should add another layer to the complex discussion of whether parasitoids contribute or not to plant fitness, as we have shown that taking only seed output into account is not enough to determine the net effect of this relationship. Other factors should be taken into consideration to properly examine the indirect effects of parasitoids on plant fitness.

## 5 CONCLUSION

The *S. latifolia–H. bicruris* interaction is usually described as parasitic, however, most studies have only focused on the net outcome of the interaction at the seed production level. Our research offers new insight into the role of parasitoids in the *S. latifolia–H. bicruris* nursery pollination system. The presence of a braconid ectoparasitoid wasp can increase seed output of the host plant, making the system more stable. However, such an increase in seed density has a negative effect on *S. latifolia* survival and flower production, and therefore, we should consider whether this increase in seed output is indeed beneficial to plant fitness. These results emphasize the need to focus on different measures of fitness when studying pollination systems and the complex relationship between natural enemies and host plants.

## CONFLICT OF INTEREST

The authors declare no conflicts of interest.

## AUTHOR CONTRIBUTIONS

CVC and TSH conceived and designed the work. CVC performed fieldwork, data collection, analysis and interpretation, and wrote the manuscript. TSH provided critical revision of the manuscript.

## Supporting information

Figure S1

Figure S2

Figure S3

## Data Availability

The data reported in this article are available in the Dryad data repository with https://doi.org/10.5061/dryad.rjdfn2z75.
