# Supplementary figures and images for: Friend or foe? A parasitic wasp shifts the cost/benefit ratio in a nursery pollination system impacting plant fitness
Source: Ecol Evol. 2020 Mar 24;10(10):4220–32. doi: 10.1002/ece3.6190 (PMC7246216; doi:10.1002/ece3.6190)

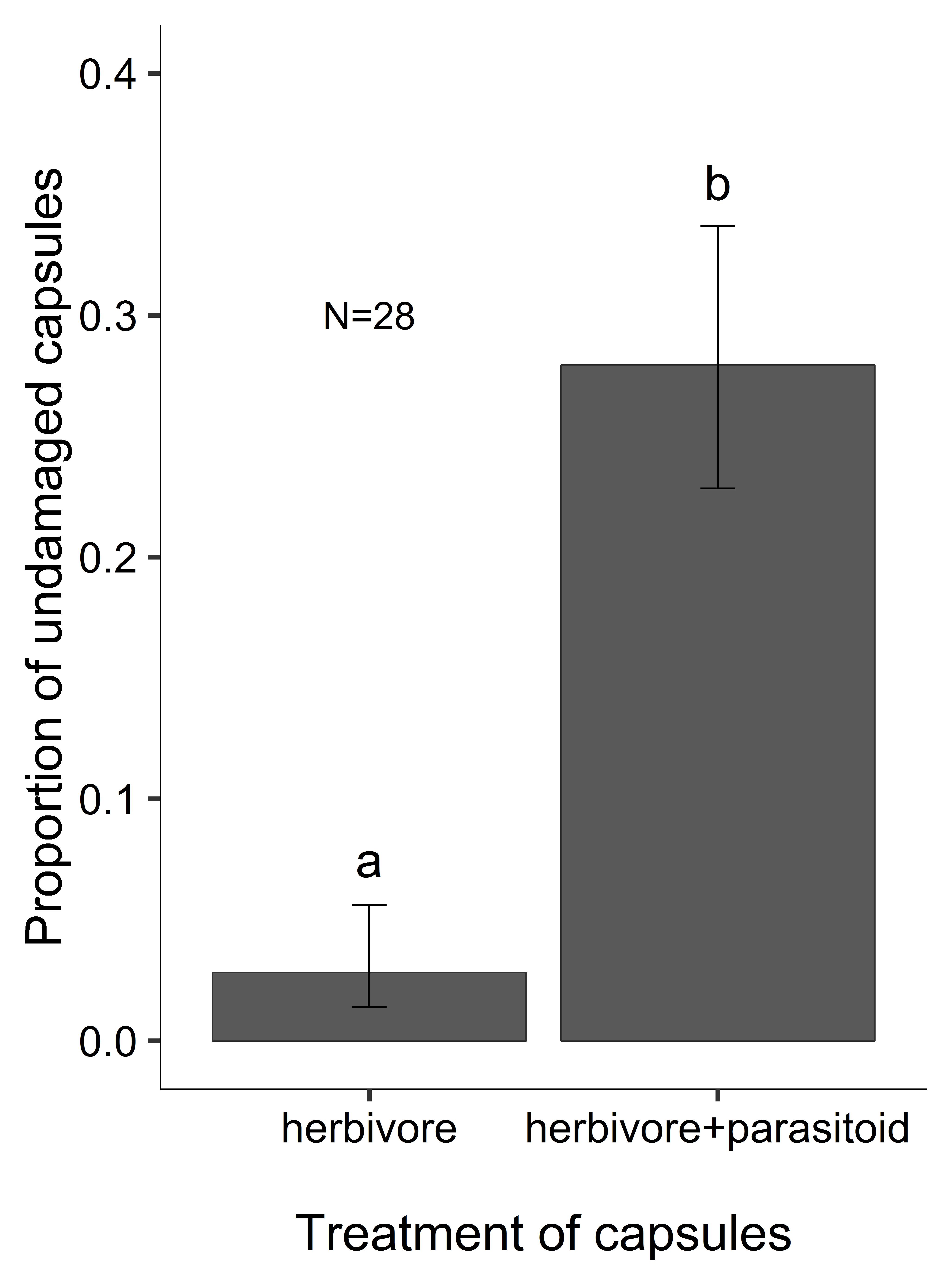

Supplement: Supplementary file 1 — Figure S1 [file ECE3-10-4220-s001.jpeg]

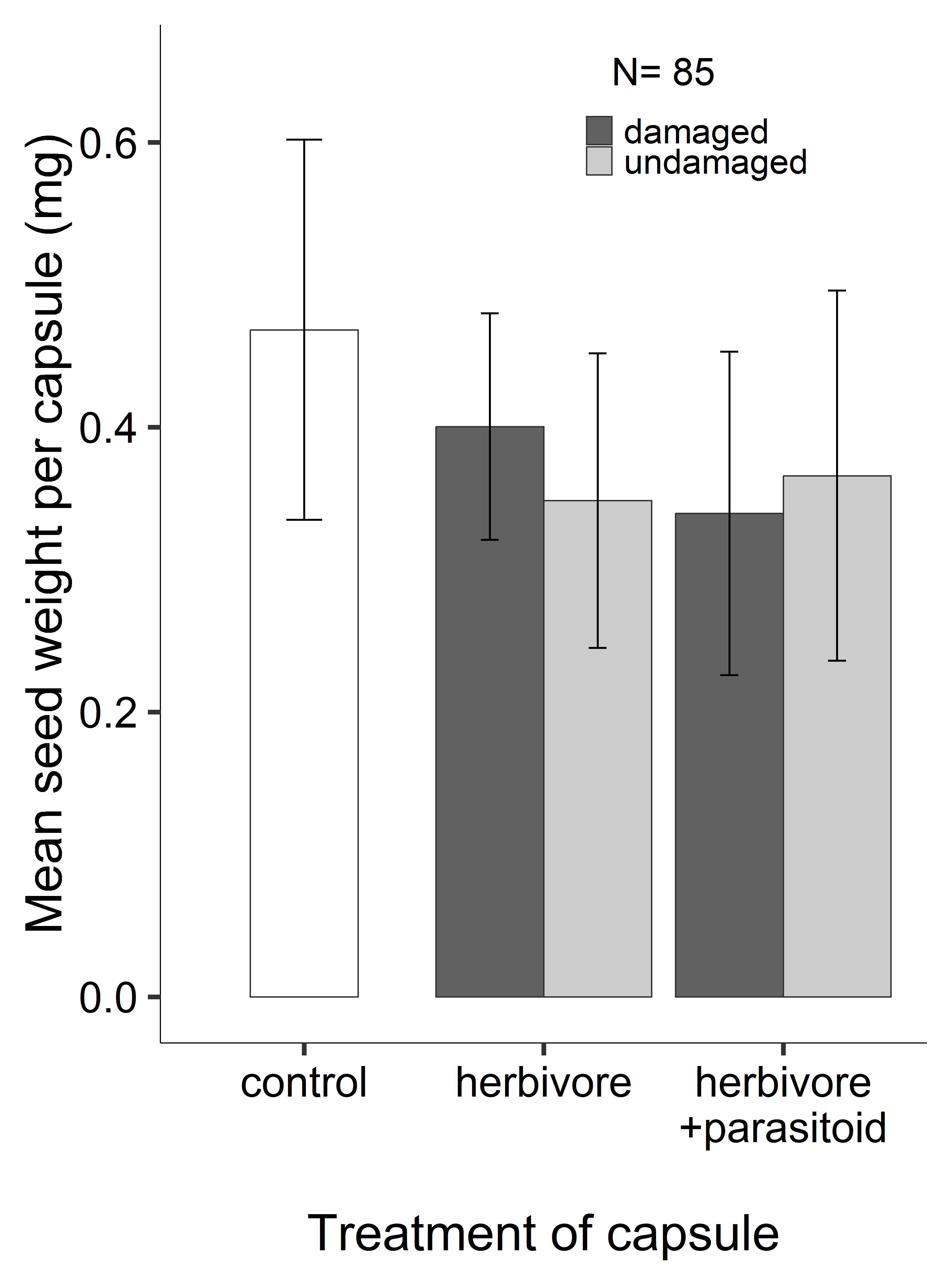

Supplement: Supplementary file 2 — Figure S2 [file ECE3-10-4220-s002.jpeg]

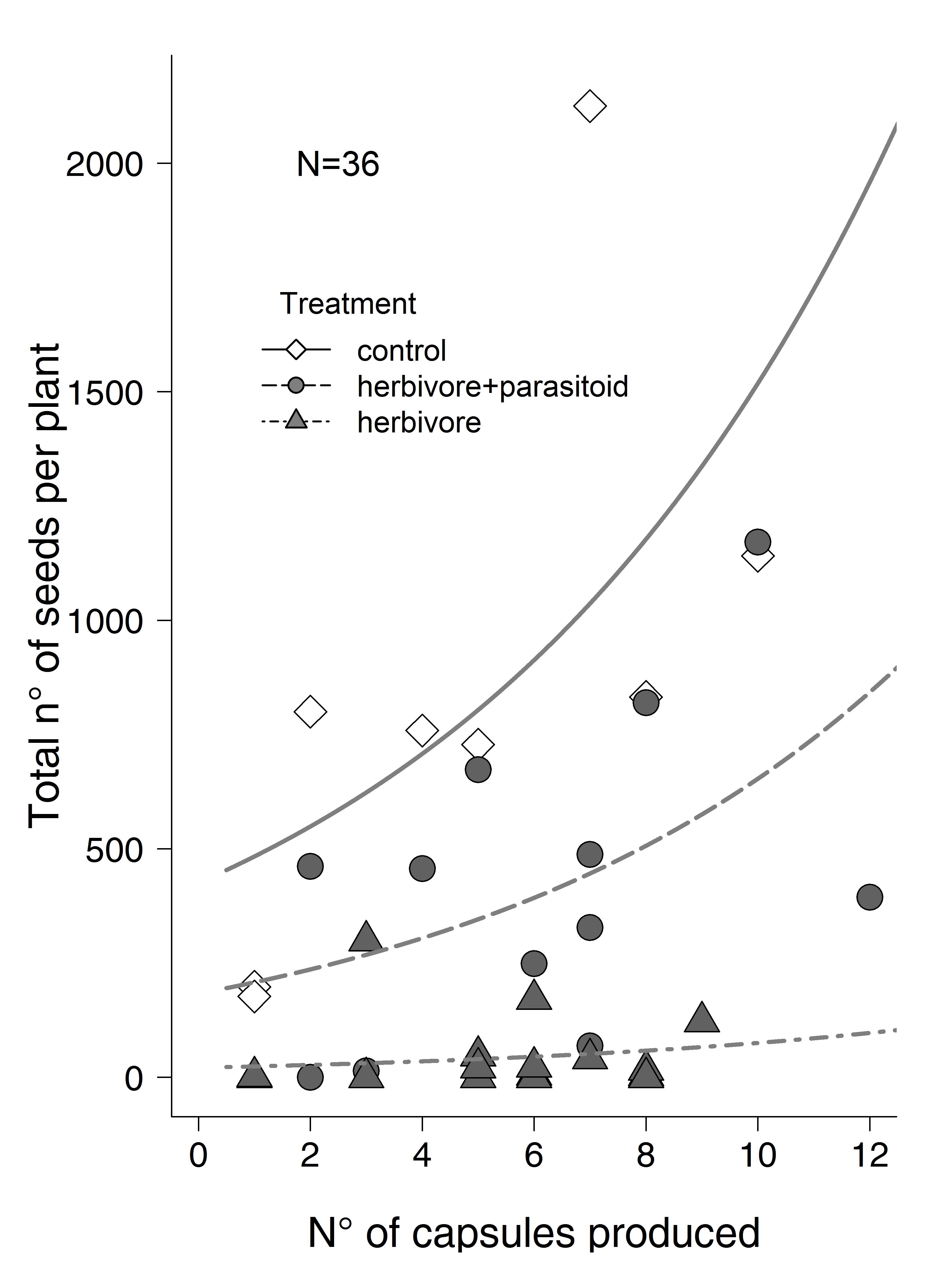

Supplement: Supplementary file 3 — Figure S3 [file ECE3-10-4220-s003.jpeg]
